# Head circumference - a useful single parameter for skull volume development in cranial growth analysis?

**DOI:** 10.1186/s13005-017-0159-8

**Published:** 2018-01-10

**Authors:** Markus Martini, Anne Klausing, Guido Lüchters, Nils Heim, Martina Messing-Jünger

**Affiliations:** 1 Department of Maxillofacial and Plastic Surgery, University of Bonn, Sigmund-Freud Str. 25, 53127 Bonn, Germany; 2 Center for Development Research (ZEF), University of Bonn, Walter-Flex-Str. 3, 53113 Bonn, Germany; 3 Department of Neurosurgery, Asklepios Children’s Hospital, Arnold-Janssen-Str. 29, 53757 Sankt Augustin, Germany; 4 Department of Oral, Maxillofacial and Plastic Surgery, University of Bonn, Welschnonnenstraße 17, D – 53111 Bonn, Germany

**Keywords:** Head circumference, Validity, Ear-to-ear measurement, Skull volume, 3d scan, Cranial growth

## Abstract

**Background:**

The measurement of maximal head circumference is a standard procedure in the examination of childrens’ cranial growth and brain development. The objective of the study was to evaluate the validity of maximal head circumference to cranial volume in the first year of life using a new method which includes ear-to-ear over the head distance and maximal cranial length measurement.

**Methods:**

3D surface scans for cranial volume assessment were conducted in this method comparison study of 44 healthy Caucasian children (29 male, 15 female) at the ages of 4 and 12 months.

**Results:**

Cranial volume increased from measurements made at 4 months to 12 months of age by an average of 1174 ± 106 to 1579 ± 79 ml. Maximal cranial circumference increased from 43.4 ± 9 cm to 46.9 ± 7 cm and the ear-to ear measurement increased from 26.3 ± 21 cm to 31.6 ± 18 cm at the same time points. There was a monotone association between maximal head circumference (HC) and increase in volume, yet a backwards inference from maximal circumference to the volume had a predictive value of only 78% (adjusted R^2^). Including the additional measurement of distance from ear to ear strengthened the ability of the model to predict the true value attained to 90%. The addition of the parameter skull length appeared to be negligible.

**Conclusion:**

The results demonstrate that for a distinct improvement in the evaluation of a physiological cranial volume development, the additional measurement of the ear-to ear distance using a measuring tape is expedient, and, especially for cases with pathological skull changes, such as craniosynostosis, ought to be conducted.

## Background

The measurement of maximal head circumference ([HC] or occipito-frontal circumference [OFC]) has been a standard procedure in the examination of childrens’ cranial growth and brain development for decades [1–4]. It is a quick, simple and economic screening method without the danger of exposure to radiation. Early detection of pathological changes are ascertained with this method. Normative data for pediatric cranial circumference and braincase volume are of multidisciplinary interest. In addition to its primary importance for differential diagnosis and therapy decisions for neurosurgical, maxillofacial- and plastic surgery, [5, 6], as well as for anthropological study of evolution [7, 8], these measurements are of immense importance to pediatric doctors and neurologists [9–13]. The collection of exact cranial volume data and anthropometric parameters is, for this reason, the subject of countless studies [14–19]. Improvements in cranial volume measurement methods rely increasingly on 3D databases. This type of data acquisition can occur in a semi-automatic manner using CT [20–22] or MRT segmentation, or, most recently, via 3D photography in combination with traditional methods of measurement [18, 23–25].

The goal of this study was to examine whether head circumference measurement alone is a good predictor of cranial volume, and whether the addition of head length and head height measurements increase the predictability of skull volume. Such additional measures included the ear-to-ear distance over the vertex to be measured for the skull height calculation as well as the head length over the top of the head point. Since cranial growth in the time between birth and the 12th month of life is the strongest [5], this evaluation focused on this period.

## Methods

Approval for the study was obtained from the local Ethic Committee of the Medicine faculty of the University of Bonn. The study was performed at the Department of Maxillofacial and Plastic Surgery at the University of Bonn and 44 healthy 4-month-old Caucasian children (29 male, 15 female) who had an unremarkable general medical history, normal course of pregnancy and unremarkable head form were included. Assessments were conducted between the ages of 4 months and 12 months from 2014 to 2016 and included a single 3D optical image scan of every child’s head without follow up.

First, 3D optical image scans of the cranium and facial surface, with the help of an optical 3D sensor (3D–Shape®, Erlangen, Deutschland). These data were triangulated and fused using Software Slim3D (3D–Shape®, Erlangen, Deutschland). After converting to a STL- format, cephalometric analysis of the data followed with the help of Software Onyx Ceph™ (Image Instruments GmbH, Chemnitz, Deutschland). Several reference parameters were identified for each patient’s cranium using Onyx Ceph™ including: three medians (Glabella [Gl], Opistocranion [Oc], the point at the top of the head [ToH]), and two bilateral (Preauricular [Pa], Infraorbital [Or]) soft tissue reference points. The Preauricular and Infraorbital points defined the horizontal plane (H), in accordance with the commonly used Frankfort horizontal plane.

After generation of the 3D data set and voxelization, intracranial volume was calculated based on the total sum of all voxels located within the space between the vertex and the angularized cranial base plane (H).

**Fig. 1:**
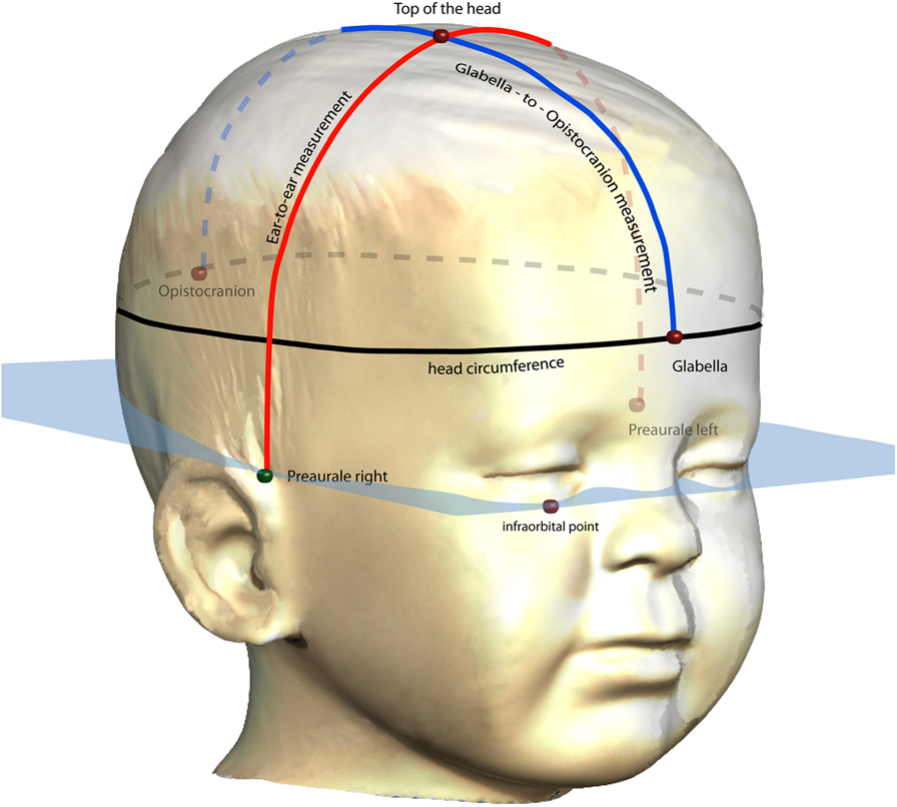
3D scan - Ear-to-ear measurement (CH), maximal head circumference (HC) and glabella-to-opistocranion measurement (CL)

Beside the maximal head circumference (HC) the cranial length (CL) from glabella to opistocranion (Gl-ToH-Oc) and the cranial height (CH), measured from cranial ear base to ear base on the contralateral side (Pa-ToH-Pa = ear-to-ear measurement; EtEm; Fig. 1) were determined using the software Onyx Ceph. Regarding the sample size the suggestion of Babyak and Rothman were followed by taking 10 to 15 observations per predictor variable (HC, CL, CH) to avoid overfitting in a multiple regression i.e. a too heavy influence by random error in the data [26, 27]. Statistical analysis was conducted using STATA 14.2 (College Station, Texas, USA), which included Pearson correlation, multiple linear regression, likelihood ratio tests, and Bland-Altman plots. Means and standard deviations are given and effect sizes are reported as partial eta^2^.

## Results

**Fig. 2:**
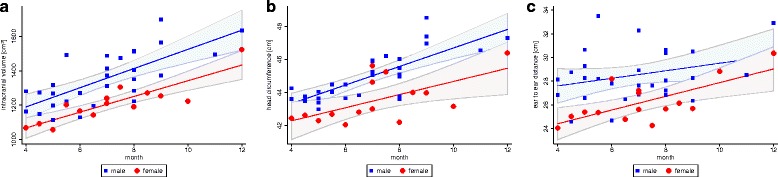
Cranial growth development in the first year of life for the parameters **a**) intracranial volume, **b**) head circumference (HC) and **c**) cranial height (CH, ear-to ear measurement over top of the head). Linear regression and 95% Confidence Intervals for girls and boys

**Fig. 3:**
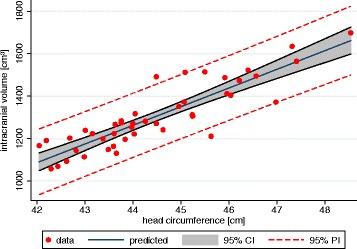
Head circumference (HC) and head volume; 95% Confidence Interval and 95% Prediction Interval

The average cranial volume for all children during the course of this study expanded from 1174 ± 106 ml (4 months) to 1579 ± 79 ml (12 months). The average intracranial volume growth among the 29 boys (1351 ± 155 ml) was larger than that of the 15 girls (1213 ± 113 ml). In the same period, maximum cranial circumference increased from 43.4 ± 9 cm to 46.9 ± 7 cm, the cranial length increased from 23.6 ± 13 cm to 25.3 ± 13 cm and the ear-to-ear measurement increased from 26.3 ± 21 cm to 31.6 ± 18 cm (Fig. 2). The maximal cranial circumference and measured volumes showed statistically significant linear correlations across all children (Pearson *r* = 0.8828; *p* = 0.000). For any given cranial circumference, 78% (R^2^) of the volume variability was explained by the model (Fig. 3).

To examine the question of whether the predictiveness can be improved by the addition of further parameters, various models were compared. It was assumed that cranial volume at the base of the skull approximates the volume of a half ellipsoid. Hence, a spherical volume calculation was made based on the ear-to-ear measurements as well as the length-girth measurement, analogue to earlier studies [28–30]. The mathematically determined cranial volumes using HC, CL and CH were compared with the voxel-based cranial volume calculation made by the software program OnyxCeph using Bland-Altman plots. These showed no clear differences in the degree of agreement of the cranial volumes between the two measurement methods. Variabilities using the two methods were also equivalently large.

Next, the predictiveness of three different multiple linear regression models were compared. First, Model A included head circumference (HC), cranial height (CH) and cranial length (CL). This model achieved highly accurate volume correspondence of 90% (adjusted R^2^). The average variance inflation factor (VIF) of 1.5 (range 1.4–1.7) eliminated the issue of collinearity. Statistically significant effects were shown for the predictors maximal circumference (*p* = 0.000) and ear-to-ear distance (*p* = 0.000). Cranial length (Gl-ToH-O), however, showed no statistically significant effect (*p* = 0.907). After a z-transformation, the maximal cranial circumference proved to be the most influential variable (beta = 0.69), followed by cranial height (beta = 0.40) and cranial length (beta = −0.007). This was also reflected by the differences in effect size quantified as partial eta^2^ (HC: 74%; CH: 54%; CL: 0.03%;).

**Fig. 4:**
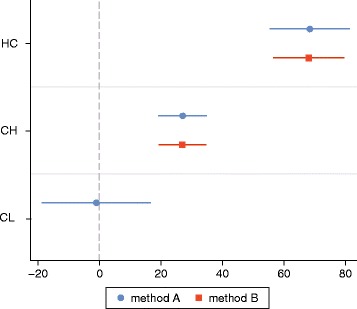
Estimated coefficients and their 95 confidence intervals of 3 measure variables (Method A), resp. 2 measure variables (Method B). according to Kastellec & Leoni [62]

Further, a reduced model based on head circumference and cranial height (Model B: HC and CH), was compared to Model A (HC, CH and CL) using a likelihood ratio test. This yielded no significant difference in predictiveness of calculated volume (B vs. A, LR: *p* = 0.902). Hence, the addition of CL had no effect on predictive value. Sex was then added as a predictor (Model C: HC, CH, Sex), which, in turn, rendered no increase in explanatory power (B vs. C, LR: *p* = 0.135). Figure 4 and Table 1 moreover show that estimated coefficients did not significantly differ in the two models. According to the principle of parsimony (Occam’s razor), Model B with the variables head circumference and ear-to-ear measurement should be preferred, since both Model A and B had an adjusted R^2^ von 90% (Table 1).

**Table 1:** Impact of 3 measure variables (Method A: head circumference [HC], cranial height [CH], cranial length [CL], 2 measure variables (Method B: HC, CH) and with added gender and age (Method C); n.s. not significant; * *p* < 0.05; ** *p* < 0.01; *** *p* < 0.001

Variable	Method A	Method B	Method C
HC	68.40***	68.07***	70.25***
CH	27.03***	26.96 ***	28.86***
CL	−1.03 n.s.		
sex			−26.87 n.s.
Intercept	−2463.02***	−2471.68***	−2603.73***
adj. R^2^	0.89	0.90	0.90

To calculate the expected cranial volume with a given HC and CL, coefficients and absolute terms were derived from linear regression model and transferred to the formula as follows: Vol. (cm3) ≙ 68 · HC + 27 · CL - 2472.

## Discussion

In the first 2 years of life the infant skull experiences its greatest structural and geometric change [31]. Intracranial volume doubles during the first 6–9 months of life [5], and increases by another 20% in the subsequent 6 months [5]. A significant positive correlation between brain volume and cranial circumference has been demonstrated by postmortem studies and CT examinations of deceased newborns [32, 33], in line with MRI studies of older children [4]. For this reason, the head circumference measure (HC) is a recognized, well-established screening parameter for intracranial volume [34–36]. This measure should, however, not be accepted without reservation, since maximal head circumference primarily reflects expansion of the base of the skull [22, 29, 37, 38].

Estimating skull volume is based, on the one hand, on country-specific HC growth reference charts, which are periodically updated [10, 13]. On the other, a wide variety of specific craniometric ratios attempt to estimate the change in skull volume and make allowances for brain configuration [29, 39]. Further, early on Buda et al. [37] pointed out that the HC in children with non-normally shaped skulls is not a valid indicator of cranial volume [37]. Skull morphology appears too complex to be represented via any single parameter, according to Marcus et al. [21], in contrast to Rijken [40]. Our own examinations of healthy children showed invalidity in the relation between HC and cranial volume (Fig. 3). The relationship was monotonously linear, yet it was not completely reliable, and showed small skull volumes for large HCs and vice versa, in line with Treit [11]. This can not be explained merely by sex-specific differences in skull form in which girls have shorter and broader skulls compared to boys [29]. At the end of the exponential skull growth phase at the age of 2 years up to the 6th year of life, the attained HC gained high reliability with *r* = 0.93 according to Rollins [10], a reliability that is reached in this study only after the addition of two further parameters (cranial height and length) for the age range 4–12 months.

Likewise, as mentioned above, the lack of validity of maximal head circumference for estimating skull volume is problematic when referencing norm values, regardless of which pathological group is used for comparison. One problem for intracranial volume determination is the lack of adequate reference material and normative age- and sex-adapted control groups based on the same evaluation procedures [41]. Even now, the most commonly referenced skull volume estimation method dates from the early 1960s which utilizes a two-dimensional radiological dataset and mathematical calculations based on the assumption of a proximal spherical volumetric relationship to estimate skull volume [42]. This estimation technique has found application by numerous authors [22, 28, 29, 38, 43] and including additional usage of a multiplier for 2D radiographic pictures [37, 42, 44]. However, the reliability 2D skull image evaluation is very limited due to inadequate reproducibility [45–47] and this method is not commensurate with modern standards of analysis. Moreover aside from country-specific living standards [13] cohort analyses show that the average HC is larger now than it was 50 years ago [29, 48]. Hence, a current comparison of HC in the literature with volume data that are even additional 10 years older warrants, at the very least, an age correction. Generally the reference data are based on segmentation of CT or MRI scans [5, 11, 14, 22] or 3D optical surface scans of healthy children [24, 25, 49]. Based on these findings, a critical debate followed regarding older publications [22, 50–52]. Recently Tenhagen [53] and Van Lindert et al. [54] compared these three different techniques and endorsed the optical 3D scan method due to its many advantages.

**Fig. 5:**
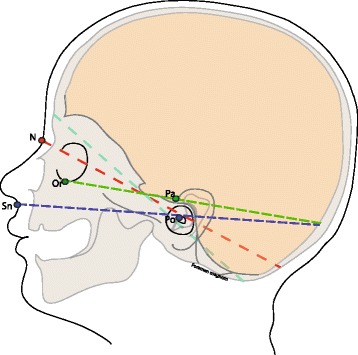
Infant cranium laterally with different h-plane angulations. N = nasion, Sn = subnasal, Or = Infra-orbitalpoint, Po = Porion; Pa = Preaural; green line – Frankfort Horizontal plane

Intracranial volume calculation based on CT-scans uses the Cavalieri principle: the cranial volume is calculated as the sum of the surface products taking into account the CT layer thickness cranial of the foramen magnum to the vertex [5, 14, 15, 23, 41, 55]. The axial layers in sequential CTs are generally aligned with the osseous frontobase and are, therefore, valid for intracranial volume detection. Modern spiral CTs even allow a multiplanar reconstruction with free H-plane referencing. Analysis software for modern 3D photogrammetry also enables free angulation of the caudal layers for volume calculations from the sum of the individual volume elements between the triangle network of the Vertex – surface data set and the specified cranial base layer (Fig. 5).

Thus, 3D photogrammetry as employed by Meyer-Marcotty et al. (analogous to MRI examinations by Tenhagen, [53]) used a caudal bounded layer through the reference points of both tragi and nasion to calculate normal volume [24]. In contrast, Seeberger [25] set this further caudal under the nose, defined via the subnasal point. Tenhagen’s intention in using a steep angle of the layer was to account for the specialness of occipital bossing in patients with scaphocephaly. They rejected the widely recognized Frankfort horizontal plane in favor of the nasion as a reference point, as Acer et al. did as well [15]. In selecting the subnasal point as a reference instead of the nasion, it should be taken into consideration that the intracranial volume estimates take large parts of the mid-face into account. Hence, Seeberger’s values are commensurately larger than those of Meyer-Marcotty et al. (with 100.32 cm^3^ at the age of 6 months and 112.05 cm^3^ at the age of 12 months). It can be problematic that, depending on the quality of the 3D laser scan image, the tragus point may be difficult to identify. In this study, therefore, the preaural point was chosen instead to define the Frankfort horizontal plane, since it is consistently easy to identify the cranial base of the ear as a reference point, and easily measurable with a tape measure for clinical examinations. Generally, the fact that the precision of the validity of the intracranial volume varies depending on the selected layer and the individual inclination of the skull base should be considered.

3D photography and CT-analysis were combined by Toma et al. [23] in their skull form analysis in children with scaphocephaly. In addition to the Cavalieri principle for volume calculation, a lot-based cranial height measurement (auricular head height: Vertex to Frankfort horizontal plane) was also used, among others parameters, to distinguish pathology from normal. As the authors point out with regret within the text of their article, such a comparison was not possible for cranial height for lack of norm values. This absence of data is due, on the one hand, to the danger of radiation exposure during CT scan for subjects, which also renders this method inappropriate for routine measurement.

On the other hand, there is also limited availability of special cephalometric measurement devices. In the clinical context, quantification of cranial measures is conducted with such instruments such as a craniometer, head spanner or anthropometric calipers. With the help of a craniometer, maximal cranial length (glabella-opistocranion) and maximal cranial width (euryon-euryon) measured through the head-center can be directly measured and the cranial volume can be determined [28, 56]. Indices such as the auricular head height via head spanner or cranial width [57] and cranial height measurements [39, 58] with the help of the spreading caliper of Hrdlička are only available in special centers and norm values with sufficiently large samples are hardly possible to generate.

Further, the possibility of 3D photocephalometry is not available to every investigator. As this study based on 3D surface scanning shows, just using a tape measure to measure to parameters enables calculation of a good approximation to the true intracranial volume. The method introduced here attained the same correlation factor (0.91) as that of 3D Photogrammetry with CT [59]. The volumes measured in this study concurred with those of the 3D surface-scan studies of Meyer-Marcotty and Seeberger regarding the 6 and 12-month evaluations of Caucasian children (see Table 2). These volumes were, however, distinctly above those of the CT based investigations by Toma [23], Abbott [14] and Sgouros [22]. The ear-to-ear measurements as well as the HC-measurements were, on the whole, slightly larger than those reported in Hou et al. for one-year-old Chinese children with 48 cm versus 47 cm and 33 cm versus 27 cm, respectively [60], whereby the HC data in this study corresponded to the percentile curves of German children in the normal range.

**Table 2:** Volume measurement (ml) according to age 1(6 months) and age 2 (12 months), relevant sample size and imaging method. In case that only graphics were presented instead of numerical values, the figures were reconstructed from these graphics using the software Digitizelt 2.2 (Braunschweig, Germany; Table 2)

	Imaging Method	Number	Age 1	Volume	Number	Age 2	Volume
Treit 2016 [11]	MRT	15	6 ± 1	1145 ± 113	22	12 ± 1	1239 ± 112
Lichtenberg 1960 [42]	X-ray		7 ± 1	920 ± 136		10 ± 1	990 ± 118
Toma 2010 [23]	CT		5–6	799		11 ± 2	997
Abbott 2000 [14]	CT	63	6 ± 1	853 ± 134		12 ± 1	1079 ± 72
Sgouros 1999 [22]	CT		6 ± 1	829 ± 104		12 ± 1	1026 ± 52
Meyer-Marcotty 2014 [24]	3D–Scan	52	6 ± 0.5	1229 ± 100	52	12 ± 0.5	1460 ± 112
This study 2017	3D–Scan	8	6 ± 0.5	1228 ± 116	3	12 ± 0.5	1551 ± 74
Seeberger 2016 [25]	3D–Scan	246	0–6	1336 ± 207	301	7–12	1527 ± 168

The visual imaging-based measurement methods a) cranial height in the form of ear-to-ear distance over the vertex, as well as b) the cranial length, measured as the distance from glabella to external occipital protuberance over the Vertex, which have been described in the literature [60, 61], were examined here for their validity with regard to volume calculation. The use of these measures (CH and CL) in addition to HC assessed with a measuring tape, decisively raise the predictive power of cranial volume of the children in the first year of life from 78% to 90%, whereby the ear-to-ear measurement is of particular relevance. This is independent of age or sex (Table 1). Hence, in daily clinical practice the predictive value of HC and CH are sufficiently high. Dolichocephalic and turicephalic head shapes can also be detected quickly, easily and validly in children with putatively normal skull shapes merely using a measuring tape, and the skull shape can be specified quantitatively as well. As far as we know, this is the first demonstration of the relationship between volume and measuring tape measurements.

There are several limitations inherent in our study. The database of this study with 44 children ranging in age from 4 to 12 months is too small to derive normative data, and requires a more extensive investigation. In addition, in as much as further studies are based on 3D photography, which reference planes should be used to optimally determine the approximate true intracranial volume needs to be explored. On the whole, the scan-based volume estimates are necessarily larger than those of real intracranial volumes, since they include in the thickness of skin, hair, cranial vault and cerebrospinal fluid space: These estimates, therefore, must lie above estimates any based on CT and autopsy findings.

## Conclusion

These results demonstrate that a clear improvement is made to the assessment of a physiological cranial volume development in children up to 12 months by the mere addition of ear-to-ear distance by means of a measuring tape, in addition to the HC. This is particularly useful for detecting pathological cranial changes as in micro- or macrocephaly or for more complex conditions, such as craniosynostoses.

## Abbreviations

CH: Cranial height
CL: Cranial length
Gl: Glabella
H: Horizontal plane
HC: Maximal head circumference,
Oc: Opistocranion
OFC: Occipito-frontal circumference
Or: Infraorbital
Pa: Preauricular
ToH: Top of the head
VIF: Variance inflation factor
